# Joint effect of hepatic steatosis index and triglyceride glucose index on cardio-renal outcomes: a real-world study

**DOI:** 10.1007/s40200-026-01937-0

**Published:** 2026-05-09

**Authors:** Yun Shen, Lizheng Shi, Vivian Fonseca, Elizabeth Nauman, Eboni Price-Haywood, Gang Hu

**Affiliations:** 1 Pennington Biomedical Research Center, Baton Rouge, LA USA; 2 Celia Scott Weatherhead School of Public Health and Tropical Medicine, Tulane University, New Orleans, LA USA; 3 School of Medicine, Tulane University, New Orleans, LA USA; 4 Louisiana Public Health Institute, New Orleans, LA USA; 5 Ochsner Clinical School - University of Queensland, New Orleans, LA USA; 6 Pennington Biomedical Research Center, Baton Rouge, LA 70808 USA

**Keywords:** Hepatic steatosis index, Triglyceride glucose index, Cardio-renal outcomes

## Abstract

**Objective:**

Hepatic steatosis index (HSI) and triglyceride glucose (TyG) index are emerging markers associated with metabolic health and cardiovascular risk. Their combined effects on cardio-renal outcomes remain poorly understood. This study evaluated the joint effect of HSI and TyG index on cardio-renal outcomes in a real-world population.

**Methods:**

We analyzed 16,109 participants, stratified by tertiles of TyG index and HSI groups (≤ 36 vs. >36). Cardio-renal outcomes included composite major adverse cardiovascular events (MACE: cerebrovascular events, coronary artery disease, heart failure, myocardial infarction) and renal events (eGFR decline ≥ 50%, end-stage kidney disease [ESKD], proteinuria). Hazard ratios (HRs) were calculated using multivariable Cox proportional hazards models adjusted for demographics and clinical covariates.

**Results:**

Higher TyG tertiles were associated with a significantly increased risk of composite cardio-renal outcomes (HR for tertile 3 vs. tertile 1: 1.58; 95% CI: 1.37–1.83). Subgroup analysis showed that individuals with HSI > 36 and TyG tertile 3 had the highest risks for composite MACE (HR: 1.62; 95% CI: 1.34–1.96) and composite renal outcomes (HR: 3.13; 95% CI: 2.10–4.68) compared with other subgroups. Both higher TyG and HSI levels showed a particularly strong association with proteinuria (HR for HSI > 36 and TyG tertile 3: 3.69; 95% CI: 2.26–6.02).

**Conclusions:**

The combined assessment of HSI and TyG index provides a valuable tool for identifying individuals at high risk for cardio-renal outcomes. These findings highlighted the importance of metabolic markers in predicting complications and underscore the need for early interventions targeting these populations.

**Supplementary Information:**

The online version contains supplementary material available at 10.1007/s40200-026-01937-0.

## Introduction

Cardiovascular disease and chronic kidney disease are leading causes of morbidity and mortality worldwide and frequently co‑occur on a shared pathobiologic axis of insulin resistance, adiposity, dyslipidemia, and low‑grade inflammation [1–3]. Despite improvements in traditional risk stratification, a substantial proportion of events arise among individuals without overt diabetes or hypertension, underscoring the need for simple, reproducible biomarkers that capture early metabolic dysfunction and refine prediction of cardio‑renal outcomes [4, 5].

The hepatic steatosis index (HSI) is a noninvasive algorithm that integrates routinely available clinical variables including body mass index and serum aminotransferases, with adjustments for sex and diabetes status, to estimate the likelihood of hepatic steatosis [6]. Hepatic fat accumulation is tightly linked to systemic insulin resistance, atherogenic dyslipidemia, endothelial dysfunction, and kidney injury; accordingly, elevated HSI has been associated with incident type 2 diabetes, cardiovascular disease, and chronic kidney disease in population‑based studies [7–11]. As a surrogate of steatotic liver disease, HSI offers an accessible way to capture a hepatic facet of metabolic risk in clinical and epidemiologic settings. The triglyceride‑glucose (TyG) index is another inexpensive surrogate of insulin resistance that correlates with clamp‑based measures and with homeostatic model assessment [12]. Higher TyG values have been linked to incident diabetes, coronary events, heart failure, and renal decline across diverse populations [13, 14]. By reflecting the combined burden of hypertriglyceridemia and dysglycemia, TyG complements HSI and may index a distinct, extrahepatic dimension of metabolic injury [15–17].

Although HSI and TyG have each been individually related to adverse outcomes, their joint effects on cardio‑renal risk remain poorly characterized. From a mechanistic perspective, coexistence of hepatic steatosis and insulin resistance could amplify lipotoxicity, oxidative stress, and pro‑inflammatory signaling, thereby accelerating atherosclerosis and glomerular injury. A combined assessment may therefore improve risk discrimination beyond either marker alone or help identify high‑risk phenotypes amenable to early intervention. To address this gap, we evaluated the joint association of HSI and TyG index with composite cardiovascular and renal outcomes in a large, real‑world cohort of adults.

## Methods

### Study design and population

Data for this study were obtained from the electronic medical records (EMRs) including both patients with and without type 2 diabetes accessed through 3 Louisiana health systems in Research Action for Health Network (REACHnet), a clinical Research Network within PCORnet, which supports patient‑centered outcomes research using real‑world clinical data [18, 19]. The dataset included EMRs for the study cohort from January 1, 2019 to December 31, 2023. Eligibility required ≥ 12 months of observable data. Participants also showed ongoing care engagement, defined as ≥ 1 routine outpatient visit or prescription refill within the 12 months preceding either health‑plan disenrollment or the study end date, whichever occurred first. A unique global identifier was used to link records across the healthcare providers to avoid the duplication of individual patients in the pooled dataset. The study and analysis plan were approved by the Institutional Review Board of Pennington Biomedical Research Center (2019-032-PBRC). All research procedures were conducted in accordance with the ethical principles of the Declaration of Helsinki. This was a secondary analysis of deidentified electronic medical record data, and the requirement for informed consent was waived by the Institutional Review Board.

**Fig. 1:**
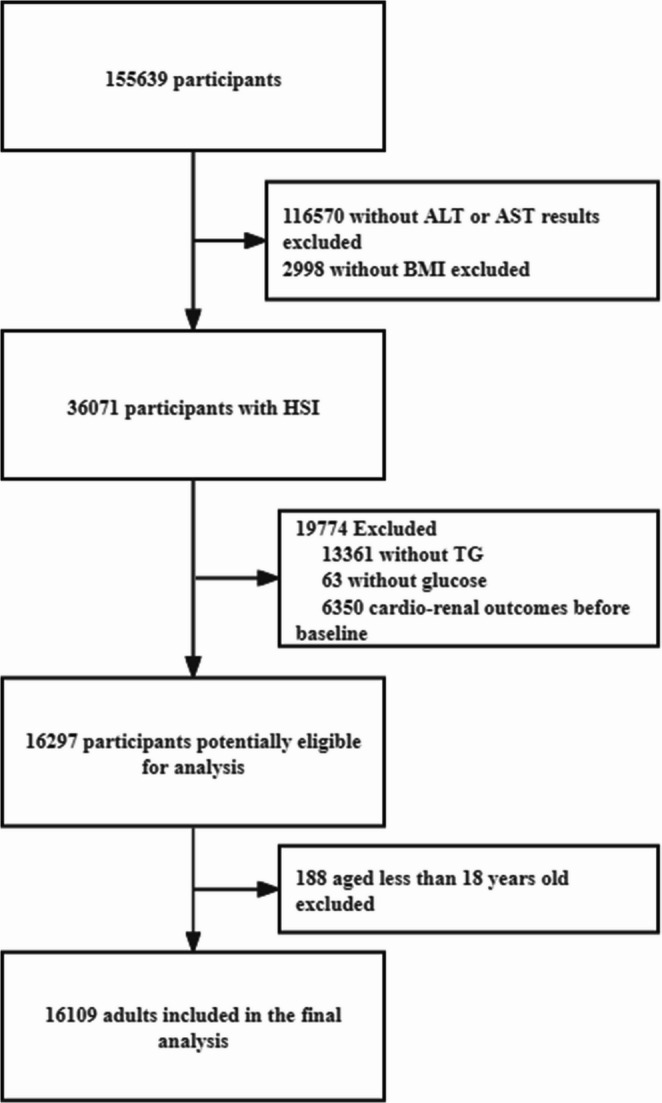
Flow chart of the study

A total of 155,639 patients between the ages of 18 and 80 years were identified and January 1, 2019 was used as the baseline date of this study. We excluded patients with no lab results and those who had developed cardio-renal outcomes of interest in this study at baseline following a complete-case strategy. The final sample for analysis included 16,109 patients, as shown in Fig. 1. To investigate for the presence of selection bias, we compared patients included in the analysis with those excluded from the analysis. Compared with patients who were excluded from the present study, the patients that were included in the analysis were of a similar age (46.2 ± 12.4 years old vs. 45.9 ± 11.8 years old), as well as similar race/ethnicity and sex (men, 44.9% vs. 45.3%) proportions in the group of patients included in our study.

## Exposure assessment and covariates

EMR data available adhere to the PCORnet Common Data Model (CDM), which standardizes data structure and content across the distributed research network [20, 21]. For this study, patient‑level data were mapped to the CDM and included demographics (date of birth, sex, race, ethnicity, and age at diabetes diagnosis); encounter dates; anthropometrics (height, weight, body mass index (BMI)); vital signs (systolic blood pressure (SBP) and diastolic blood pressure (DBP)); health behaviors (tobacco use); diagnoses with index dates; laboratory dates and results (total cholesterol, triglycerides, HDL‑cholesterol, LDL‑cholesterol, HbA1c, and serum creatinine for kidney function); and medication orders/refills (antihypertensives, glucose‑lowering agents, and lipid‑lowering therapies). Smoking status was captured from visit‑level self‑report and categorized as current, former, or never.

Hepatic steatosis index (HSI) was calculated as 8 × ALT/AST + BMI (+ 2 if type 2 diabetes yes, + 2 if female) [6]. The TyG index was calculated as Ln (fasting triglycerides [mg/dL] × fasting plasma glucose [mg/dL]/2) [22]. The estimated glomerular filtration rate (eGFR) was calculated using the Chronic Kidney Disease Epidemiology (CKD-EPI) equation [23].

A priori covariates included age, sex, race, ethnicity, and comorbidities of hypertension, diabetes, and dyslipidemia, which were defined using the International Classification of Disease (ICD) 10 codes corroborated by vitals or laboratory thresholds when applicable. Diabetes was determined by ICD-10 codes E08-E13 or random glucose levels ≥ 200 mg/dL or HbA1c ≥ 6.5%. Hypertension was determined by ICD-10 codes I10 or SBP ≥ 140mmHg or DBP ≥ 90mmHg. Dyslipidemia was determined by ICD-10 codes E78.0, E78.1, E78.2, E78.3, E78.4, and E78.5 or TG ≥ 200 mg/dL or TC ≥ 240 mg/dL or LDL ≥ 160 mg/dL or HDL < 40 mg/dL for male or HDL < 50 mg/dL for female.

### Outcomes and follow-ups

We examined cardiovascular, renal, and composite cardio‑renal outcomes using validated diagnosis codes and laboratory criteria. Major adverse cardiovascular events (MACE) were defined as composite of cerebrovascular events, coronary artery events, heart failure, ischemic stroke, and myocardial infarction. ICD code sets included cerebrovascular events (ICD‑10 I60–I69), coronary artery events (ICD‑10 I21–I25), heart failure (ICD‑10 I50), ischemic stroke (ICD‑10 I63), and myocardial infarction (ICD‑10 I21–I23). Composite renal outcomes were defined as either (a) sustained eGFR decline ≥ 50% confirmed by a subsequent eGFR value ≥ 30 days later; (b) end‑stage kidney disease (ESKD) defined as eGFR < 15 mL/min/1.73 m², initiation of dialysis, or kidney transplantation (ICD‑10 N18.5, N18.6, N19; dialysis codes ICD‑10 N18.6, Z99.2, Z49.0; transplant codes ICD‑10 Z94.0, T86.1); or (c) proteinuria (ICD‑10 R80). Composite cardio‑renal outcomes were defined as any MACE or renal endpoint.

For each outcome class, participants contributed person‑time from baseline until the first qualifying event, health‑plan disenrollment, death (if available), or December 31, 2023, whichever occurred first.

### Statistical analysis

Baseline characteristics are presented overall, by TyG tertile, and by HSI category in Table 1. Continuous variables are summarized as mean (SD) or median (IQR) as appropriate. Between‑group differences were evaluated with one‑way ANOVA or Kruskal–Wallis tests. Categorical variables were reported as counts (percent) and compared using χ² tests. Incidence was summarized using events and person‑years; where reported, incidence rates per 1,000 person‑years were computed as cases divided by person‑time with Poisson 95% CIs. For each outcome (composite and component events), we fit Cox proportional hazards models to estimate hazard ratios (HRs) and 95% confidence intervals (CIs) across TyG tertiles, using tertile 1 (T1) as the referent. Multivariable models adjusted for age, sex, race, ethnicity, and medication use of hypertension, diabetes and dyslipidemia. Covariates were selected based on univariate analysis (Supplemental Table S5). P for trend across TyG tertiles was obtained by assigning the median TyG of each tertile and modeling this ordinal score as a continuous variable. We evaluated effect modification by fatty‑liver burden using HSI stratification (≤ 36 vs. > 36) and a multiplicative TyG × HSI interaction term; P‑interaction values are reported from Wald tests. Proportional hazards were assessed using Schoenfeld residuals and log-log survival plots; if violations were detected, we used stratified Cox models or introduce time‑varying coefficients for the offending covariate. Follow‑up time accrued from baseline (index) to first event, death, loss to follow‑up, or administrative end of follow‑up, whichever came first; individuals without an event were right‑censored at their last known contact. Receiver operating characteristic (ROC) analyses were performed to compare the discriminative ability of TyG, HSI, and their combined model for predicting cardio-renal outcomes. Area under the curves (AUC) were reported. All tests were two‑sided with α = 0.05. Given a priori hypotheses for TyG and HSI, we did not adjust for multiple comparisons across secondary endpoints. All analyses were performed in R (version 4.4.3) and *P* < 0.05 was considered significant.

**Table 1:** Baseline characteristics of the study participants by tertiles of triglyceride glucose index

	Overall (*n* = 16,109)	Triglyceride glucose index			
		Tertile 1	Tertile 2	Tertile 3	*P*
		(*n* = 5,370)	(*n* = 5,370)	(*n* = 5,369)	
Triglyceride glucose index	8.51 (0.65)	7.86 (0.25)	8.44 (0.15)	9.22 (0.50)	0.001
Age, years	46.2 (12.4)	43.3 (12.7)	46.8 (12.3)	48.5 (11.7)	<0.001
Sex, n (%)					<0.001
Male	7,225 (44.9)	1,786 (33.3)	2,367 (44.1)	3,072 (57.2)	
Female	8,884 (55.1)	3,584 (66.7)	3,003 (55.9)	2,297 (42.8)	
Ethnicity, n (%)					0.007
Not Hispanic	15,341 (95.2)	5,130 (95.5)	5,136 (95.6)	5,075 (94.5)	
Hispanic	640 (3.97)	190 (3.54)	196 (3.65)	254 (4.73)	
Not known	128 (0.79)	50 (0.93)	38 (0.71)	40 (0.75)	
Race, n (%)					<0.001
American Indian or Alaska Native	64 (0.40)	23 (0.43)	23 (0.43)	18 (0.34)	
Asian	311 (1.93)	94 (1.75)	82 (1.53)	135 (2.52)	
Black or African American	2996 (18.6)	1303 (24.3)	962 (17.9)	731 (13.6)	
White	12,396 (77.0)	3,844 (71.6)	4,205 (78.3)	4,347 (81.0)	
Other	337 (2.09)	105 (1.96)	97 (1.81)	135 (2.52)	
Medication use, n (%)					
Diabetes	4,040 (25.1)	845 (15.7)	1,162 (21.6)	2,033 (37.9)	< 0.001
Hypertension	6,649 (41.3)	1,509 (28.1)	2,192 (40.8)	2,948 (54.9)	< 0.001
Dyslipidemia	7,485 (46.5)	1,400 (26.1)	2,409 (44.9)	3,676 (68.5)	< 0.001
Body mass index, kg/m^2^	30.2 (7.33)	27.7 (6.73)	30.4 (7.39)	32.5 (7.07)	<0.001
Systolic blood pressure, mm Hg	124 (15.4)	120 (14.4)	124 (14.8)	128 (15.9)	<0.001
Diastolic blood pressure, mm Hg	77.7 (9.86)	75.6 (9.66)	77.7 (9.46)	79.7 (10.0)	<0.001
Smoking, n (%)					<0.001
Never	14,366 (89.2)	4,928 (91.8)	4,795 (89.3)	4,643 (86.5)	
Former	543 (3.37)	136 (2.53)	168 (3.13)	239 (4.45)	
Current	559 (3.47)	126 (2.35)	194 (3.61)	239 (4.45)	
Unknown	641 (3.98)	180 (3.35)	213 (3.97)	248 (4.62)	
Total cholesterol, mg/dL	192 (40.6)	179 (34.2)	193 (37.0)	203 (45.8)	<0.001
Triglycerides, mg/dL	119 (119)	59.7 (13.7)	98.4 (18.3)	199 (178)	<0.001
HDL cholesterol, mg/dL	54.7 (16.0)	62.3 (16.3)	55.3 (14.8)	46.4 (12.5)	<0.001
LDL cholesterol, mg/dL	110 (37.0)	102 (30.5)	114 (37.4)	112 (39.8)	0.001
Glucose, mg/dL	103 (43.0)	89.9 (11.6)	96.8 (14.3)	122 (68.2)	0.000
HbA1c, %	5.71 (1.33)	5.22 (0.51)	5.40 (0.58)	6.31 (1.82)	<0.001

Data were in mean (SD) or number (%)

## Results

### Baseline characteristics

The analytic cohort included 16,109 adults, divided into TyG index tertiles (Sample size, T1 = 5,370; T2 = 5,370; T3 = 5,369, respectively, Table 1). Across increasing TyG tertiles, participants were older, a greater proportion were male, and the prevalences of diabetes, hypertension and hyperlipidemia were higher (all *P*_trend_ < 0.001). Adiposity and cardiometabolic parameters showed consistent adverse gradients with higher TyG index including BMI, SBP, DBP, fasting glucose, HbA1c and triglycerides, whereas HDL-c decreased significantly (all *P*_trend_ ≤ 0.001).

We also presented the baseline characteristics by HSI ≤ 36 (*n* = 5,638) and HSI > 36 (*n* = 10,471) (Supplemental Table 1). Compared with HSI ≤ 36, those with HSI > 36 were slightly older (46.9 vs. 45.1 years), more often male (47.5% vs. 39.9%), and had substantially higher prevalences of diabetes (32.0% vs. 12.1%), hypertension (50.6% vs. 24.0%), and hyperlipidemia (52.9% vs. 34.5%). BMI (33.7 vs. 23.8 kg/m²), SBP/DBP, fasting glucose, HbA1c, and triglycerides were higher and HDL‑C lower in HSI > 36 (all *P* ≤ 0.001).

## Event occurrence by TyG tertiles and by HSI

Over follow‑up, cardiorenal and cardiovascular event proportions increased stepwise across TyG tertiles (Supplemental Table 2). The composite cardiorenal outcome rose from 4.84% (T1) to 12.5% (T3); composite MACE from 4.28% to 9.67%; coronary events from 2.79% to 6.59%; heart failure from 0.89% to 2.05%; ischemic stroke from 1.01% to 2.40%; and myocardial infarction from 0.39% to 1.34% (all *P* < 0.001). Composite renal events increased from 0.88% to 4.08%, driven largely by proteinuria, eGFR decline ≥ 50% and ESKD also increased with TyG (both *P* ≤ 0.003). Similar trends could be seen when the incidence of the cardio-renal outcomes was reported by HSI groups (Supplemental Table 3).

## Associations of TyG index with cardio‑renal outcomes

In multivariable Cox models adjusted for age, sex, race, ethnicity, and hypertension (Table 2), higher TyG was associated with greater risk across most outcomes. Compared with T1, adjusted hazard ratios (aHR [95% CI]) for T2 and T3 were: composite cardiorenal, 1.21 (1.04–1.42) and 1.58 (1.37–1.83) (*P*_trend_ < 0.001); composite MACE, 1.20 (1.01–1.41) and 1.34 (1.14–1.58) (*P*_trend_ < 0.001); coronary events, 1.24 (1.02–1.52) and 1.29 (1.06–1.56) (P‑trend = 0.018); heart failure, 1.07 (0.74–1.56) and 1.48 (1.04–2.10) (*P*_trend_ = 0.017); ischemic stroke, 1.02 (0.71–1.46) and 1.61 (1.16–2.24) (P‑trend = 0.001); and myocardial infarction, 2.05 (1.24–3.38) and 1.91 (1.17–3.15) (*P*_trend_ = 0.030). For renal outcomes, associations were strongest: composite renal, 1.32 (0.91–1.90) and 3.21 (2.32–4.44) (*P*_trend_ < 0.001); eGFR decline ≥ 50%, 0.63 (0.22–1.84) and 2.07 (0.88–4.82) (P‑trend = 0.041); ESKD, 1.66 (0.56–4.89) and 4.25 (1.60–11.3) (*P* < 0.001); and proteinuria, 1.53 (0.95–2.47) and 4.29 (2.80–6.58) (*P* < 0.001).

**Table 2:** Associations between tertiles of triglyceride glucose index with cardio-renal outcomes

	Adjusted hazard ratio (95% confidence interval)			*P* for trend
	TyG Tertile 1	TyG Tertile 2	TyG Tertile 3	
Composite cardio-renal outcome	1.0	1.21 (1.04–1.42)	1.58 (1.37–1.83)	< 0.001
Composite MACE	1.0	1.20 (1.01–1.41)	1.34 (1.14–1.58)	< 0.001
Coronary artery event	1.0	1.24 (1.02–1.52)	1.29 (1.06–1.56)	0.018
Heart failure	1.0	1.07 (0.74–1.56)	1.48 (1.04–2.10)	0.017
Ischemic stroke	1.0	1.02 (0.71–1.46)	1.61 (1.16–2.24)	0.001
Myocardial infarction	1.0	2.05 (1.24–3.38)	1.91 (1.17–3.15)	0.030
Composite renal outcomes	1.0	1.32 (0.91–1.90)	3.21 (2.32–4.44)	< 0.001
eGFR decline ≥ 50%	1.0	0.63 (0.22–1.84)	2.07 (0.88–4.82)	0.041
ESKD	1.0	1.66 (0.56–4.89)	4.25 (1.60–11.3)	< 0.001
Proteinuria	1.0	1.53 (0.95–2.47)	4.29 (2.80–6.58)	< 0.001

Adjusted hazard ratio (95% CI) was adjusted for age, sex, race, ethnicity, and medication use of diabetes, hypertension and dyslipidemia

*MACE* major adverse cardiovascular events

ESKD was defined by eGFR < 15 mL/min/1·73m^2^, requirement of dialysis or kidney transplantation

### Interaction between TyG index and HSI on cardio‑renal outcomes

In Table 3, formal tests of interaction demonstrated heterogeneity by HSI for ischemic stroke (*P* for interaction = 0.037) and myocardial infarction (*P* for interaction = 0.032), but not for the other endpoints (*P* for interaction ≥ 0.152). In HSI‑stratified analyses, the TyG gradient for ischemic stroke was pronounced at HSI ≤ 36 (T3 aHR 2.93, 95% CI 1.71–5.03 in one model set; aHR 2.76, 1.59–4.79 with a *P* for trend < 0.001 in the companion table) and attenuated at HSI > 36 (T3 aHR 1.47, 0.92–2.35; *P* for trend = 0.066). For myocardial infarction, the association was stronger at HSI > 36 (T2/T3 aHRs 3.40 [1.53–7.57] and 3.42 [1.56–7.51]; *P* for trend = 0.008), with no significant association at HSI ≤ 36. For composite renal outcomes, TyG was positively associated in both HSI strata, without significant interaction (*P* for interaction = 0.152). Similar trends were observed across TyG tertiles in different HSI groups on both MACE and renal outcomes (Table 4). Subgroup analysis by age, sex and race further showed similar results (Supplemental Table S4).

**Table 3:** Associations between tertiles of triglyceride glucose index and HSI with cardio-renal outcomes

	HSI≤36			HSI>36			*P* for interaction
	TyG Tertile 1	TyG Tertile 2	TyG Tertile 3	TyG Tertile 1	TyG Tertile 2	TyG Tertile 3	
Composite cardio-renal outcome	1.0	1.22 (0.95-1.56)	1.52 (1.16-1.98)	0.98 (0.77-1.26)	1.19 (0.96-1.48)	1.58 (1.29-1.93)	0.630
Composite MACE	1.0	1.21 (0.93-1.57)	1.43 (1.08-1.90)	0.96 (0.74-1.25)	1.16 (0.92-1.45)	1.29 (1.04-1.60)	0.627
Coronary artery event	1.0	1.15 (0.84-1.57)	1.07 (0.75-1.52)	0.87 (0.63-1.20)	1.16 (0.89-1.52)	1.23 (0.95-1.58)	0.345
Heart failure	1.0	1.35 (0.72-2.51)	1.59 (0.82-3.09)	1.19 (0.66-2.14)	1.12 (0.65-1.94)	1.64 (0.99-2.72)	0.873
Ischemic stroke	1.0	1.21 (0.67-2.18)	2.93 (1.71-5.03)	1.10 (0.64-1.90)	1.02 (0.61-1.69)	1.47 (0.92-2.35)	0.037
Myocardial infarction	1.0	1.34 (0.65-2.75)	0.50 (0.16-1.52)	0.44 (0.17-1.09)	1.47 (0.80-2.71)	1.49 (0.83-2.69)	0.032
Composite renal outcomes	1.0	1.57 (0.84-2.92)	2.36 (1.25-4.46)	1.23 (0.68-2.23)	1.46 (0.85-2.51)	3.86 (2.36-6.32)	0.152
eGFR decline ≥50%^*^	1.0	/	1.08 (0.19-6.00)	0.71 (0.17-2.92)	0.72 (0.20-2.60)	1.84 (0.60-5.62)	0.219
ESKD	1.0	1.31 (0.18-9.36)	5.09 (0.96-26.8)	0.99 (0.16-6.00)	1.76 (0.37-8.42)	4.08 (0.94-17.7)	0.794
Proteinuria	1.0	2.05 (0.79-5.31)	2.95 (1.11-7.82)	1.92 (0.80-4.61)	2.46 (1.09-5.57)	7.21 (3.34-15.6)	0.310

Adjusted hazard ratio (95% CI) was adjusted for age, sex, race, ethnicity, and medication use of diabetes, hypertension and dyslipidemia

*MACE* major adverse cardiovascular events

ESKD was defined by eGFR < 15 mL/min/1·73m^2^, requirement of dialysis or kidney transplantation

^*^Insufficient case number in HSI ≤ 36 with TyG Tertile 2 group

**Table 4:** Associations between tertiles of triglyceride glucose index with cardio-renal outcomes in two HSI groups

	HSI ≤ 36				HSI > 36			
	TyG Tertile 1	TyG Tertile 2	TyG Tertile 3	*P* for trend	TyG Tertile 1	TyG Tertile 2	TyG Tertile 3	*P* for trend
Composite cardio-renal outcome	1.0	1.20 (0.93–1.54)	1.47 (1.12–0.93)	0.005	1.0	1.23 (1.00-1.50)	1.62 (1.34–1.96)	< 0.001
Composite MACE	1.0	1.20 (0.92–1.56)	1.40 (1.05–1.87)	0.019	1.0	1.21 (0.97–1.50)	1.35 (1.10–1.66)	0.004
Coronary artery event	1.0	1.15 (0.84–1.58)	1.08 (0.75–1.56)	0.581	1.0	1.34 (1.02–1.77)	1.41 (1.08–1.84)	0.019
Heart failure	1.0	1.35 (0.72–2.52)	1.50 (0.77–2.94)	0.224	1.0	0.95 (0.59–1.52)	1.40 (0.91–2.15)	0.052
Ischemic stroke	1.0	1.18 (0.66–2.13)	2.76 (1.59–4.79)	< 0.001	1.0	0.93 (0.59–1.47)	1.36 (0.89–2.06)	0.066
Myocardial infarction	1.0	1.34 (0.65–2.77)	0.51 (0.17–1.58)	0.427	1.0	3.40 (1.53–7.57)	3.42 (1.56–7.51)	0.008
Composite renal outcomes	1.0	1.55 (0.83–2.91)	2.27 (1.18–4.36)	0.014	1.0	1.19 (0.76–1.88)	3.13 (2.10–4.68)	< 0.001
eGFR decline ≥ 50%^*^	1.0	/	0.83 (0.14–4.84)	0.596	1.0	1.06 (0.30–3.80)	2.81 (0.92–8.59)	0.026
ESKD	1.0	1.30 (0.18–9.38)	5.10 (0.92–28.1)	0.051	1.0	1.81 (0.48–6.86)	4.20 (1.23–14.3)	0.006
Proteinuria	1.0	2.28 (0.87–5.97)	3.34 (1.20–9.27)	0.018	1.0	1.27 (0.73–2.22)	3.69 (2.26–6.02)	< 0.001

Adjusted hazard ratio (95% CI) was adjusted for age, sex, race, ethnicity, and medication use of diabetes, hypertension and dyslipidemia

*MACE* major adverse cardiovascular events

ESKD was defined by eGFR < 15 mL/min/1·73m^2^, requirement of dialysis or kidney transplantation

^*^Insufficient case number in HSI ≤ 36 with TyG Tertile 2 group

In ROC analysis (Supplemental Table S6), for composite cardio-renal outcomes, the AUC was 0.63 for TyG, 0.57 for HSI, and 0.64 for the combined TyG-HSI model. Similar patterns were observed for composite MACE and its components. For renal outcomes, discrimination was stronger. The AUC for composite renal outcomes was 0.71 for TyG, 0.65 for HSI, and 0.72 for the combined model. Notably, the combined TyG-HSI model showed the highest AUC values for ESKD (0.71) and proteinuria (0.74).

## Discussion

In this large observational cohort, higher TyG index was associated with a graded increase in cardiorenal risk, with particularly strong associations for renal outcomes (composite renal events, proteinuria, and ESKD). The risk of composite MACE and several cardiovascular components (coronary events, heart failure, ischemic stroke, and myocardial infarction) also rose across TyG tertiles after adjustment for age, sex, race, ethnicity, and hypertension. These findings are consistent with prior evidence that insulin resistance and dysmetabolic states contribute to both macrovascular and microvascular injury and extend that work by demonstrating robust associations in a contemporary, multi-system US health-care network. Together, they support the concept that TyG captures a clinically relevant cardiometabolic risk phenotype that is not fully reflected by traditional risk factors alone.

Hepatic steatosis indexed by the HSI both tracked with higher event rates and modified select TyG-outcome associations. The TyG index is a pragmatic surrogate of insulin resistance and HSI is also a validated proxy for hepatic steatosis. Together they capture complementary aspects of dysmetabolism, lipotoxicity, hyperglycemia, and liver fat burden, that plausibly drive both macrovascular and microvascular injury. Prior studies typically evaluate TyG or hepatic steatosis measures in isolation. We quantify their combined association with outcomes and show that the TyG‑HSI phenotype identifies a materially higher‑risk group. The disproportionately stronger associations for renal outcomes (including proteinuria) align with the concept that insulin resistance [24] and hepatic fat promote glomerular hyperfiltration [25], endothelial dysfunction, and tubular injury [26], yielding earlier and larger relative risks for kidney endpoints than for atherosclerotic events within similar follow‑up windows. The HSI‑specific findings suggest biologically nuanced risk profiles. The more pronounced TyG gradient for myocardial infarction at HSI > 36 may reflect the confluence of hepatic steatosis, atherogenic dyslipidemia, and systemic inflammation that favors plaque vulnerability. In contrast, the stronger TyG-ischemic stroke gradient at HSI ≤ 36 could indicate pathways less dependent on frank steatosis (e.g., hypertension‑mediated small‑vessel disease or prothrombotic milieu). That said, differences in sample size and event counts by stratum, along with multiple comparisons, warrant cautious interpretation of these interactions. The coexistence of these abnormalities may amplify endothelial dysfunction, plaque progression, and plaque instability, thereby predisposing to coronary thrombosis and myocardial infarction. In this context, the TyG HSI combination may identify different phenotypes with different disease outcomes. Nevertheless, these interaction findings should be interpreted with caution. Event numbers in some TyG HSI strata were limited, particularly for specific cardiovascular subtypes, leading to wide confidence intervals. In addition, multiple interaction tests were conducted, increasing the possibility of chance findings. Therefore, while the observed heterogeneity is biologically plausible and hypothesis generating, confirmation in independent cohorts with larger numbers of events and longer follow up is warranted.

A key rationale for focusing on TyG and HSI in this study is their pragmatic compatibility with EMR-based risk stratification. Both indices can be computed using routine clinical data like fasting triglycerides, fasting glucose, aminotransferases, BMI, and simple sex and diabetes indicators without the need for imaging, specialized tests, or additional cost. This makes them attractive for large-scale deployment in data networks and health systems. At the same time, we recognize that TyG and HSI are not yet now widely used in clinical practice and established markers such as FIB-4 (for liver fibrosis) and risk engines such as BRAVO or PREVENT are more familiar to clinicians. Importantly, our dataset did not consistently capture the platelet counts and harmonized timing of AST/ALT measurements required to compute FIB-4 reliably across the cohort, and fasting insulin was not available to calculate HOMA-IR or related insulin resistance indices. As a result, we were unable to perform head-to-head comparisons of TyG + HSI versus TyG + FIB-4 or versus broader CVD risk scores, and our findings should be viewed as demonstrating prognostic relevance of an easily computed TyG-HSI phenotype rather than as proof of superiority over established tools. Future work in datasets with richer laboratory panels should explicitly compare the incremental predictive value of TyG and HSI relative to, and in combination with, FIB-4, BRAVO, PREVENT, and other widely used indices.

Beyond statistical significance, our findings have practical implications for clinical care and for health system risk stratification. Both TyG and HSI are derived from routinely collected laboratory and anthropometric measures and can be automatically calculated from electronic medical records without additional cost, imaging, or patient burden. This makes them particularly well suited for large scale implementation in real world settings, including automated flagging of individuals who may benefit from earlier evaluation and closer follow up. In our cohort, individuals in the highest TyG tertile had substantially higher risk of composite cardio renal outcomes, and the risk was most pronounced among those who also had HSI greater than 36. This combined TyG HSI phenotype likely captures a metabolically high risk profile characterized by insulin resistance and hepatic steatosis burden, and it identified a subgroup with markedly elevated renal risk and increased cardiovascular risk compared with other strata. From a clinical perspective, this joint assessment could be used as a simple first pass screen to prioritize more intensive cardio renal risk assessment in routine practice. Individuals with both elevated TyG and HSI may warrant earlier and more frequent kidney surveillance, including urine albumin screening and closer monitoring of estimated glomerular filtration rate trajectories, given the strong associations observed for renal outcomes and proteinuria. In parallel, this phenotype could prompt more proactive management of modifiable cardiometabolic risk factors, such as tighter control of blood pressure and glycemia, and reinforcement of lifestyle interventions targeting adiposity and insulin resistance. In settings where multiple competing priorities exist, a readily computable TyG HSI signal embedded in the EMR could help clinicians identify patients who may benefit most from targeted counseling and risk factor optimization.

Strengths of our study include a large sample, consistent definitions of exposures and outcomes, analysis of time‑to‑event endpoints with clear person‑time denominators and a prespecified set of covariates applied uniformly across outcomes. TyG and HSI were calculated from routinely collected clinical data, demonstrating the feasibility of embedding these indices in real-world data environments without additional testing burden. The parallel presentation of composite and component endpoints, stratification by HSI, and the provision of event counts and person‑years by TyG×HSI strata enhance transparency and interpretability. However, several limitations should be considered when interpreting these findings. First, this was an observational analysis of electronic medical record data, and causal inference cannot be made. Although we adjusted for key demographic and clinical covariates, residual confounding may persist due to unmeasured or incompletely captured factors, including validated medication exposure, treatment intensity, medication adherence, diet, physical activity, socioeconomic factors, and health care access. In addition, TyG and HSI were assessed at baseline and may change over time; such within person variability could attenuate associations and may limit clinical interpretation based on a single measurement. Importantly, although TyG and HSI are attractive because they are low cost and easily derived from routinely collected clinical data, they are not sufficient to replace established cardiovascular and renal risk assessment tools. Our results support the prognostic relevance of a readily computable TyG HSI phenotype, but they do not define treatment thresholds or provide evidence that clinical decision making should be based on these indices alone. Instead, TyG and HSI should be viewed as adjunctive markers that may help identify high risk metabolic phenotypes and complement routine risk evaluation, particularly in settings where early metabolic dysfunction is not well captured by conventional risk factors. Before implementation in clinical practice, the utility of TyG and HSI for screening and risk stratification should be validated in external cohorts with different demographic and clinical profiles and in health systems beyond the current multi system network. Future studies should also examine whether adding TyG and HSI meaningfully improves discrimination and reclassification beyond established prediction engines, and whether interventions that reduce insulin resistance and liver fat translate into lower cardio renal event rates in prospective trials.

In conclusion, higher TyG identifies individuals at elevated risk for both cardiovascular and, especially, renal events, and this risk is further contextualized by hepatic steatosis burden. The combination of TyG and HIS, two measures readily available from routine clinical data may help prioritize preventive strategies for cardiorenal protection. Future work should incorporate richer confounding control (e.g., medication use, kidney function, lifestyle), repeated measures of metabolic indices, and external validation in diverse populations to define actionable thresholds and to test whether modifying TyG‑related pathways mitigates the observed risks.

## Supplementary Information

Below is the link to the electronic supplementary material.

Supplementary Material 1

## Data Availability

The data that supports the findings of this study are available from the corresponding author upon reasonable request.
