# Supplementary material for: Joint effect of hepatic steatosis index and triglyceride glucose index on cardio-renal outcomes: a real-world study
Source: J Diabetes Metab Disord. 2026 May 9;25(1):132. doi: 10.1007/s40200-026-01937-0 (PMC13157377; doi:10.1007/s40200-026-01937-0)
Supplement: Supplementary file 1 — Supplementary Material 1 [file 40200_2026_1937_MOESM1_ESM.docx]

Supplemental Table S1. Baseline characteristics of the study participants by HSI group

|  | **HSI ≤36** | **HIS>36** | **p value** |
| --- | --- | --- | --- |
|  | **(n=5,638)** | **(n=10,471)** |  |
| HSI | 31.9 (2.87) | 45.2 (7.45) | <0.001 |
| Age, years | 45.1 (13.3) | 46.9 (11.9) | <0.001 |
| Sex, n (%) |  |  | <0.001 |
| Male | 2,248 (39.9) | 4,977 (47.5) |  |
| Female | 3,390 (60.1) | 5,494 (52.5) |  |
| Ethnicity, n (%) |  |  | 0.003 |
| Hispanic | 5,351 (94.9) | 9,990 (95.4) |  |
| Not Hispanic | 224 (3.97) | 416 (3.97) |  |
| Not known | 63 (1.12) | 65 (0.62) |  |
| Race, n (%) |  |  | <0.001 |
| American Indian or Alaska Native | 16 (0.28) | 48 (0.46) |  |
| Asian | 162 (2.87) | 149 (1.42) |  |
| Black or African American | 745 (13.2) | 2,251 (21.5) |  |
| White | 4,589 (81.4) | 7,807 (74.6) |  |
| Other | 123 (2.18) | 214 (2.04) |  |
| Medication use, n (%) |  |  |  |
| Diabetes | 685 (12.1) | 3,355 (32.0) | <0.001 |
| Hypertension | 1,353 (24.0) | 5,296 (50.6) | <0.001 |
| Dyslipidemia | 1,947 (34.5) | 5,538 (52.9) | <0.001 |
| BMI, kg/m^2^ | 23.8 (2.71) | 33.7 (6.66) | <0.001 |
| Systolic blood pressure, mm Hg | 120 (14.6) | 127 (15.2) | <0.001 |
| Diastolic blood pressure, mm Hg | 74.8 (9.63) | 79.2 (9.63) | <0.001 |
| Smoking, n (%) |  |  | 0.007 |
| Never | 5,079 (90.1) | 9,287 (88.7) |  |
| Former | 156 (2.77) | 387 (3.70) |  |
| Current | 195 (3.46) | 364 (3.48) |  |
| Unknown | 208 (3.69) | 433 (4.14) |  |
| Total cholesterol, mg/dL | 191 (38.0) | 192 (41.9) | 0.023 |
| Triglycerides, mg/dL | 90.8 (56.2) | 134 (139) | <0.001 |
| HDL cholesterol, mg/dL | 62.3 (17.0) | 50.6 (13.8) | <0.001 |
| LDL cholesterol, mg/dL | 107 (36.3) | 112 (37.3) | 0.089 |
| Glucose, mg/dL | 93.7 (19.3) | 108 (50.8) | <0.001 |
| HbA1c, % | 5.26 (0.72) | 5.89 (1.46) | <0.001 |

Data were in mean (SD) or number (%).

Supplemental Table S2. Incidence of the cardio-renal outcomes by tertiles of triglyceride glucose index

|  | **Overall (n=16109)** | **Triglyceride glucose index** | | |  |
| --- | --- | --- | --- | --- | --- |
|  |  | **Tertile 1** | **Tertile 2** | **Tertile 3** | **p value** |
|  |  | **(n=5,370)** | **(n=5,370)** | **(n=5,369)** |  |
| **Composite cardio-renal outcome** | 1322 (8.21) | 253 (4.71) | 406 (7.56) | 663 (12.4) | <0.001 |
| **Composite MACE** | 1089 (6.76) | 222 (4.13) | 359 (6.69) | 508 (9.46) | <0.001 |
| Coronary artery event, n (%) | 769 (4.77) | 150 (2.79) | 265 (4.93) | 354 (6.59) | <0.001 |
| Heart failure, n (%) | 224 (1.39) | 48 (0.89) | 66 (1.23) | 110 (2.05) | <0.001 |
| Ischemic stroke, n (%) | 252 (1.56) | 54 (1.01) | 69 (1.28) | 129 (2.40) | <0.001 |
| Myocardial infarction, n (%) | 153 (0.95) | 21 (0.39) | 60 (1.12) | 72 (1.34) | <0.001 |
| **Composite renal outcomes** | 292 (1.81) | 37 (0.69) | 58 (1.08) | 197 (3.67) | <0.001 |
| eGFR decline ≥50%, n (%) | 35 (0.22) | 8 (0.15) | 6 (0.11) | 21 (0.39) | 0.003 |
| ESKD, n (%) | 45 (0.28) | 5 (0.09) | 10 (0.19) | 30 (0.56) | <0.001 |
| Proteinuria, n (%) | 233 (1.45) | 26 (0.48) | 48 (0.89) | 159 (2.96) | <0.001 |

MACE: major adverse cardiovascular events.

ESKD was defined by eGFR <15 mL/min/1·73m^2^, requirement of dialysis or kidney transplantation.

Supplemental Table S3. Incidence of the cardio-renal outcomes by HSI

|  | **HSI ≤36** | **HIS>36** | **p value** |
| --- | --- | --- | --- |
|  | **(n=5,638)** | **(n=10,471)** |  |
| **Composite cardio-renal outcome** | 337 (5.98) | 985 (9.41) | <0.001 |
| **Composite MACE^a^** | 303 (5.37) | 786 (7.51) | <0.001 |
| Coronary artery event, n (%) | 209 (3.71) | 560 (5.35) | <0.001 |
| Heart failure, n (%) | 57 (1.01) | 167 (1.59) | 0.003 |
| Ischemic stroke, n (%) | 78 (1.38) | 174 (1.66) | 0.197 |
| Myocardial infarction, n (%) | 34 (0.60) | 119 (1.14) | 0.001 |
| **Composite renal outcomes** | 42 (0.74) | 250 (2.39) | <0.001 |
| eGFR decline ≥50%, n (%) | 6 (0.11) | 29 (0.28) | 0.041 |
| ESKD, n (%) | 9 (0.16) | 36 (0.34) | 0.050 |
| Proteinuria, n (%) | 28 (0.50) | 205 (1.96) | <0.001 |

MACE: major adverse cardiovascular events.

ESKD was defined by eGFR <15 mL/min/1·73m^2^, requirement of dialysis or kidney transplantation

Supplemental Table S4. Sex and age subgroup analysis of cardio-renal composite outcomes by different TyG and HIS.

|  | **HSI≤36** | | |  | **HSI>36** | | | | | ***P* for interaction** | |
| --- | --- | --- | --- | --- | --- | --- | --- | --- | --- | --- | --- |
|  | **TyG Tertile 1** | **TyG Tertile 2** | **TyG Tertile 3** |  | **TyG Tertile 1** | **TyG Tertile 2** | | | **TyG Tertile 3** |  |  |
| **Sex** |  |  |  |  |  | |  |  | |  |  |
| Male | 1.00 | 1.01 (0.73, 1.40) | 1.25 (0.90, 1.75) |  | 0.89 (0.64, 1.23) | | 0.97 (0.73, 1.28) | 1.27 (0.98, 1.64) | |  | 0.20 |
| Female | 1.00 | 1.46 (0.98, 2.19) | 2.06 (1.30, 3.25) |  | 1.09 (0.74, 1.60) | | 1.57 (1.12, 2.20) | 2.22 (1.60, 3.08) | |  | 0.64 |
| Age |  |  |  |  |  | |  |  | |  |  |
| >=65 years old | 1.00 | 1.46 (0.74, 2.87) | 1.17 (0.57, 2.41) |  | 0.93 (0.43, 2.02) | | 1.36 (0.73, 2.52) | 1.71 (0.96, 3.03) | |  | 0.05 |
| <65 years old | 1.00 | 1.28 (0.97, 1.69) | 1.92 (1.43, 2.56) |  | 0.97 (0.74, 1.26) | | 1.24 (0.98, 1.56) | 1.67 (1.34, 2.08) | |  | 0.37 |
| Race |  |  |  |  |  | |  |  | |  |  |
| Black or African American | 1.00 | 1.12 (0.58, 2.17) | 1.98 (1.06, 3.70) |  | 0.69 (0.40, 1.17) | | 1.29 (0.80, 2.08) | 1.76 (1.10, 2.82) | |  | 0.14 |
| White | 1.00 | 1.23 (0.93, 1.63) | 1.49 (1.10, 2.02) |  | 1.09 (0.82, 1.46) | | 1.15 (0.90, 1.47) | 1.49 (1.19, 1.88) | |  | 0.40 |
| Others | 1.00 | 0.54 (0.09, 3.30) | 1.21 (0.26, 5.53) |  | 1.81 (0.40, 8.14) | | 0.78 (0.16, 3.88) | 3.25 (0.96, 10.98) | |  | 0.63 |

Adjusted hazard ratio (95 % CI) was adjusted for age, sex, race, ethnicity, and medication use of diabetes, hypertension and dyslipidemia.

Supplemental Table S5. Univariate hazard ratios for all covariates selected into the multivariate models of cardio-renal composite outcomes.

|  | **Hazard ratios** |
| --- | --- |
| Covariates |  |
|  |  |
| Triglyceride glucose index | 1.84 (1.72, 1.96) |
| Age, years | 1.07 (1.06, 1.07) |
| Sex |  |
| Male | 1.00 |
| Female | 0.50 (0.44, 0.55) |
| Ethnicity |  |
| Not Hispanic | 1.00 |
| Hispanic | 0.82 (0.61, 1.10) |
| Race |  |
| Black or African American | 1.00 |
| White | 0.91 (0.79, 1.04) |
| Other | 0.73 (0.53, 0.99) |
| Diabetes | 3.3 (2.96, 3.67) |
| Hypertension | 4.51 (3.99, 5.11) |
| Hyperlipidemia | 4.22 (3.71, 4.81) |
| Body mass index | 1.03 (1.02, 1.03) |
| Systolic blood pressure | 1.02 (1.02, 1.03) |
| Diastolic blood pressure | 1.01 (1.01, 1.02) |
| Smoking |  |
| Never | 1.00 |
| Former | 1.55 (1.20, 1.98) |
| Current | 1.63 (1.27, 2.08) |
| Total cholesterol | 1.00 (1.00, 1.00) |
| Triglycerides | 1.00 (1.00, 1.00) |
| HDL cholesterol | 0.97 (0.97, 0.98) |
| LDL cholesterol | 0.99 (0.98, 1.00) |
| Glucose | 1.00 (1.00, 1.00) |
| HbA1c | 1.28 (1.24, 1.31) |

Supplemental Table S6. ROC analysis comparing the effect of TyG, HSI and TyG-HSI on cardio-renal outcomes.

|  | **TyG** | **HSI** | **TyG + HSI** |  |
| --- | --- | --- | --- | --- |
| **Composite cardio-renal outcome** | 0.63 (0.62, 0.65) | 0.57 (0.56, 0.59) | 0.64 (0.62, 0.65) |  |
| **Composite MACE** | 0.61 (0.60, 0.63) | 0.55 (0.54, 0.57) | 0.61 (0.60, 0.63) |  |
| Coronary artery event | 0.61 (0.60, 0.63) | 0.55 (0.53, 0.57) | 0.61 (0.59, 0.63) |  |
| Heart failure | 0.60 (0.57, 0.64) | 0.58 (0.55, 0.62) | 0.62 (0.58, 0.65) |  |
| Ischemic stroke | 0.62 (0.59, 0.66) | 0.52 (0.48, 0.55) | 0.62 (0.59, 0.66) |  |
| Myocardial infarction | 0.63 (0.58, 0.67) | 0.58 (0.54, 0.62) | 0.63 (0.58, 0.67) |  |
| **Composite renal outcomes** | 0.71 (0.68, 0.75) | 0.65 (0.62, 0.68) | 0.72 (0.69, 0.75) |  |
| eGFR decline ≥50% | 0.63 (0.53, 0.73) | 0.62 (0.54, 0.71) | 0.66 (0.57, 0.75) |  |
| ESKD | 0.69 (0.62, 0.77) | 0.60 (0.52, 0.68) | 0.71 (0.64, 0.78) |  |
| Proteinuria | 0.73 (0.69, 0.76) | 0.67 (0.64, 0.70) | 0.74 (0.70, 0.77) |  |

Area under the ROC curves with 95%CI were shown in the table.
